# Use of Clinical Trial Characteristics to Estimate Costs of New Drug Development

**DOI:** 10.1001/jamanetworkopen.2024.53275

**Published:** 2025-01-06

**Authors:** Andrew Mulcahy, Stephanie Rennane, Daniel Schwam, Reid Dickerson, Lawrence Baker, Kanaka Shetty

**Affiliations:** 1 RAND, Arlington, Virginia; 2 RAND, Santa Monica, California; 3 Now with Department of Economics, University of California, Los Angeles

## Abstract

**Importance:**

Despite their importance to patients, health, and industry, the magnitude of investments in drug research and development (R&D) remain nebulous. New policies require more granular and transparent R&D cost estimates to better balance incentives for innovation and returns to developers.

**Objective:**

To estimate per-drug R&D costs using a novel, reproduceable approach and to describe firm-level R&D costs per discrete unit of R&D activity (1 patient-month).

**Design, Setting, and Participants:**

This economic evaluation used cross-sectional data to estimate 2014 to 2019 costs per patient-month. Costs per patient-month were calculated using data from 268 US publicly traded drug developers, contributing 1311 firm-year observations, that were highest ranked by assets or market capitalization, after exclusions. Per-drug costs were calculated from all R&D activity through approval for a cohort of 38 new drugs approved by the US Food and Drug Administration in 2019. Data were analyzed from January 2022 to July 2024.

**Exposure:**

R&D activity, measured in terms of clinical trial patient-months.

**Main Outcomes and Measures:**

This study used a 2-step approach to estimate R&D costs, first allocating firm-year–level total R&D spending across similarly aggregated patient-months, and then aggregating these incremental costs to estimate drug-level R&D costs per new drug.

**Results:**

Among 268 developers assessed, 20 large firms accounted for 80.8% of all patient-months and had 27.4% lower mean and 26.7% lower median costs per patient-month compared with other firms. Each 1% increase in patient-months was associated with a 0.9% increase in R&D costs. R&D costs per new drug were highly skewed, with a lower median (IQR), at $708 million ($247 million to $1.42 billion) than mean (SD), at $1.31 ($1.92) billion, after adjusting for the cost of capital and discontinued products. Without these adjustments, direct costs per new drug were a median (IQR) of $150 ($67.6-$453) million and a mean (SD) $369 of ($684) million. While estimated R&D costs varied in sensitivity analyses, mean costs were always substantially greater than median costs.

**Conclusions and Relevance:**

This economic evaluation found median per-drug R&D costs toward the lower end of the range from prior studies, with a mean closer to the middle of the existing range despite the broad scope of included costs. These findings suggest parallel development across indications, adjustment for discontinued products, and a small number of expensive development programs are particularly important drivers of R&D costs.

## Key Points

**Question:**

What is the estimated cost to develop a new drug and how do research and development (R&D) costs vary across firms?

**Findings:**

This economic evaluation examining 268 US-traded drug developers found that, in 2019, 20 firms accounted for 80.8% of R&D activity (measured as clinical trial patient-months) and had 27.4% lower mean and 26.7% lower median costs per patient-month vs other firms. Median and mean costs per 2019 new approved drug were $708 million and $1.31 billion, respectively, after cost of capital and discontinuation adjustments.

**Meaning:**

These findings suggest that R&D costs per new drug are highly skewed, so both median and mean costs should be considered.

## Teaser

This economic evaluation estimates per-drug research and development costs and describes firm-level costs per discrete unit of research and development activity.

## Introduction

Remarkable developments in medicine, including COVID-19 treatments and cures for hepatitis C, result from investments in drug research and development (R&D). These successes have played out through a vigorous debate on the extent to which US drug price regulation—for example, Medicare negotiation^1^ for older, single-source drugs introduced by the Inflation Reduction Act (IRA)—might decrease R&D investments. The pharmaceutical industry argues lower prices will have catastrophic effects on R&D.^2^ Some prior studies have reported associations between price regulation and lower domestic R&D investment in other countries.^3,4^ The CBO’s score of the IRA projected lower Medicare prices would result in fewer new drugs over 30 years, but only by 1%.^5^ Others point to the adverse effects of high drug prices on patient access, finances, and health^6,7^ and maintain that companies would still bring important new drugs to market even at lower prices.^8^

These arguments relate to broader questions on the socially optimal level of R&D.^9,10,11^ While most agree that greater expected monetary returns incentivize R&D,^12,13,14^ the disagreement in pharmaceuticals focuses on whether the current equilibrium, where US prices for brand-name drugs are more than 200% those in other countries,^15^ leads to the optimal level and targeting of R&D investments.

Despite the centrality of drug-level R&D cost estimates to these debates, data are rarely disclosed by industry,^16^ and policymakers must rely on estimates from researchers. One oft-cited study estimates a mean R&D cost per self-originated new molecular entity (NME) at $2.8 billion (in 2018 $ US),^17^ while other recent estimates are lower ($879 million to $1.7 billion).^18,19,20^ This range reflects differences in setting and methodology,^19^ often driven by data availability considerations, and often stemming from ambiguity in the seemingly straightforward terms *new drug*, *R&D*, and *cost*. For *new drug*, studies differ in covering costs from discovery through an initial regulatory approval or beyond, eg, through subsequent approvals and postmarketing trials, with further distinctions for estimates in specific therapeutic area (eg, oncology^21^) or for certain drug candidates (eg, self-originated new drugs^17^). For *R&D*, many prior studies focus on clinical development rather than R&D broadly, quantifying clinical research activity by counts of trials by phase^22^ or enrolled patients. Studies vary in their handling of preclinical research and overhead (if included at all).^20^ On *cost*, studies often report direct costs and costs after adjustments for the cost of capital and discontinued development, with postadjustment costs often several times larger than direct costs. The source of cost information is also an important differentiator: some studies^17^ use proprietary, self-reported industry costs that cannot be validated.^23,24^ Many use cost data from a narrow sets of firms^17,21^ or drugs^21^ or rely on cost inputs and assumptions from analysts and vendors,^20^ raising important transparency and generalizability concerns.

Our study uses a novel approach to estimate R&D costs for new drugs, mitigating several of these challenges. To better account for variation in clinical research intensity and the full scope of R&D costs, we first estimate costs per patient-month using 6-year, firm-wide R&D cost and activity data from 268 drug developers. We apply these estimates to data on the number and timing of patient-months in clinical trials from the start of development through regulatory approval for a cohort of 38 NMEs approved by the US Food and Drug Administration (FDA) in 2019. Finally, we describe variation in estimates across the cohort of new drugs. While mean per-drug R&D costs are commonly reported and cited,^17,20^ we report medians and means to better characterize the underlying skewed distribution.

## Methods

This economic evaluation was determined to not involve human participants by the RAND Corporation institutional review board. This study is reported following the Consolidated Health Economic Evaluation Reporting Standards (CHEERS) reporting guideline, as applicable.^25^

### Data on Firm-Level R&D Spending and Patient-Months

We used 3 main data sources. We used the S&P Capital IQ Database to identify the largest 200 publicly traded pharmaceutical firms engaging in novel drug R&D development^26^ in terms of either assets or market capitalization, resulting in a combined 268 firms.^26^ For these firms, we abstracted 2014 to 2019 annual R&D expenses from publicly available US Securities and Exchange Commission (SEC) filings (in 2019 $ US)^27^ (eAppendix 1 in Supplement 1).

We used Citeline’s Trialtrove database, which consolidates and standardizes clinical trial data from ClinicalTrials.gov and other sources.^28^ For each phase 1 to 4 trial, Trialtrove notes the drugs and indications studied, sponsors, enrollment, key dates, and status (eg, ongoing, completed, and terminated). We used this database to derive firm-year counts of (1) patient-months, which aggregate trial-level enrolled patients across all of the firm’s clinical trials during the year, and (2) the number of new trials beginning in each year. eAppendix 1 in Supplement 1 describes our approach to identify relevant trials, impute missing data, distribute patient-months over the duration of trials, and allocate shared R&D activities to sponsors and products. After aggregation, we excluded company-year observations without R&D expenses (40 firm-years), without trials (131 firm-years), or with fewer than 100 patient-months in 1 or more years (67 firm-years, including 4 firms entirely) for a total of 264 study firms and 1311 firm-years. We assess the impact of these and other exclusion criteria in the eAppendix 2 in Supplement 1.

### Estimating Costs Per Patient-Month

To obtain a cost per patient-month, we estimated linear regressions of log-transformed, firm-year–level R&D expenses on the log-transformed patient-months counts, year-fixed effects, and a constant. We present unweighted and weighted results, using patient-month count weights to approximate industry-wide associations between patient-months and costs. Models included robust SEs adjusted for correlations within firm. We used coefficients estimated from these models to calculate the mean weighted and unweighted marginal cost per incremental patient-month.

To assess heterogeneity in this association, we present results for subsamples of firms, including a stable 2014 to 2019 panel, the top 20 firms by 2019 R&D expenditures, all firms outside the top 20, and firms with 10-K vs 20-F annual SEC filings. Separately, we estimated costs per patient-months with different trial-level inclusion criteria. Finally, we estimated other model specifications as robustness checks, including (1) adding counts of annual new trials starts to account for fixed initial costs, (2) using by-phase log-transformed patient-months, (3) omitting phase 2 trials, and (4) using separate log-transforming patient-month counts for oncology vs nononcology trials. Further details are provided in the eAppendix 2 in Supplement 1).

### Estimating R&D Costs Per New Drug

We extracted all trials with start dates prior to each drug’s initial FDA approval for a cohort of 38 NMEs approved by the FDA in 2019 (eAppendix 3 in Supplement 1). We did not restrict trials to those studying the initial approved indication. As in our estimate of costs per patient-month, we proportionally allocated patient-months for trials involving more than 1 drug or sponsor. To estimate the direct costs of drug R&D, we multiplied patient-months aggregated across applicable trials for each drug by our estimated cost per patient-month adjusted for inflation.

Firms face risks of foregone investments and discontinued R&D efforts; to account for these additional costs, we applied previously published cost of capital rates (8.1% annually^29^) and estimates of discontinuation rates by therapeutic area and phase^30^ in sequence to direct costs. In sensitivity analyses, we calculated R&D costs per new drug assuming alternative cost of capital rates, including trials still in progress at the time the drug was approved by the FDA, and under different attribution assumptions for trials with more than 1 drug or sponsor.

*P* values were 2-sided, and statistical significance was set at *P* ≤ .05. Analyses were conducted using Stata version 17 (StataCorp). Data were analyzed from January 2022 to July 2024.

## Results

### R&D Spending and Patient-Month Descriptive Statistics

Among 268 developers contributing 1311 firm-years of data, R&D spending and patient-months were concentrated in a handful of firms: in 2019, the top 20 firms accounted for 74.4% of R&D spending and 80.8% of total patient-months (Table). Mean annual R&D spending among the top 20 firms was approximately 30 times the mean among other firms in 2019 ($4.7 billion vs $140 million). The difference in total patient-month means was even larger (approximately 50-fold), suggesting potential economies of scale or a different mix of R&D activity at larger firms.

**Table.** Firm Sample and Firm-Year Descriptive Statistics

Measure	All years^a^	2014	2015	2016	2017	2018	2019
**Top 20 firms by 2019 R&D expenditures (stable panel; n = 120 firm-years)**							
R&D expense, mean (95% CI), millions, $	4812.8 (4243.0-5382.6)	5030.1 (3406.0-6654.2)	4800.9 (3401.8-6200.0)	4824.6 (3389.4-6259.7)	4672.2 (3316.1-6028.3)	4848.2 (3438.2-6258.2)	4700.9 (3396.2-6005.7)
Patient-months, mean (95% CI), thousands	595.3 (510.3-680.4)	732.8 (438.2-1027.4)	642.8 (407.4-878.2)	605.9 (407.0-804.8)	544.2 (377.4-710.9)	502.0 (343.6-660.3)	544.4 (371.6-717.3)
**Firms outside the top 20 (n = 242 firms; n = 1181 firm-years)**							
No. of firm-years (% of 244 max firms)	1191 (100)	152 (62.3)	180 (73.8)	195 (80.0)	210 (86.1)	226 (92.6)	228 (93.4)
R&D expense, mean (95% CI), millions, $	113.2 (105.3-121.2)	92.5 (71.4-113.7)	97.4 (77-117.7)	106.9 (87.2-126.7)	107.8 (89.9-125.7)	123.5 (105.7-141.2)	139.8 (119.8-159.7)
Patient-months, mean (95% CI), thousands	10.6 (9.6-11.5)	9.8 (6.9-12.7)	10.1 (7.5-12.7)	10.8 (8.3-13.3)	10.5 (8.1-12.9)	10.5 (8.4-12.5)	11.4 (9.2-13.5)
**Stable panel of firms (n = 146 firms; n = 876 firm-years)**							
R&D expense, mean (95% CI), millions, $	780.2 (648.3-912.1)	778.9 (428.0-1129.9)	763.0 (440.8-1085.3)	777.7 (452.2-1103.3)	761.9 (449.7-1074.1)	802.5 (479.0-1126.0)	797.1 (488.7-1105.4)
Patient-months, mean (95% CI), thousands	93.8 (76.2-111.4)	110.1 (53.7-166.4)	99.3 (52.0-146.5)	95.6 (53.1-138.1)	87.4 (50.3-124.6)	82.0 (47.5-116.6)	88.3 (50.7-125.9)
**All firms combined**							
No. of firm-years (% of 264 max firms)	1311 (100)	172 (65.2)	200 (75.8)	215 (81.4)	230 (87.1)	246 (93.2)	248 (93.9)
R&D expense, mean (95% CI), millions, $	586.5 (493.1-680.0)	683.8 (382.3-985.2)	627.3 (375.7-878.9)	600.6 (364.4-836.8)	558.6 (344.3-772.9)	554.8 (348.7-760.9)	531.5 (340.0-722.9)
Patient-months, mean (95% CI), thousands	71.1 (58.0-84.1)	95.1 (47.0-143.3)	84.4 (44.8-124.0)	74.9 (42.4-107.4)	64.9 (37.5-92.4)	57.8 (33.6-82.0)	59.2 (34.8-83.7)

Abbreviation: R&D, research and development.

^a^ For the reported number of firms, all years reports the number of unique firms with R&D expenses and more than 100 patient-months across all 6 years. For mean R&D expense and patient-treatment months, the all years means are calculated across all firm-year records. With 1 exception, none of the 2014 vs 2019 pairwise differences were statistically significant at *P* < .05 (the increase in patient-months for firms outside the top 20 was significant, at *P* = .002). Two large firms with only 5 years of data were excluded from the all firms combined analysis to avoid large year-on-year changes. We excluded 1 firm that otherwise would have been in the top 20 (No. 19) because it lacked data for 2014. We added the 21st ranked firm so that each of the top 20 firms contributed 6 years of data (ie, they all also contribute to the stable analysis).

In the stable panel of firms , mean R&D expenses remained relatively flat, as did patient-months over the same period, with no statistically significant differences (Table). Across all firms, mean R&D expense and patient-months decreased over time, driven by greater representation of smaller firms in 2018 (the year we used to define our sample) (Table).

**Figure 1.**
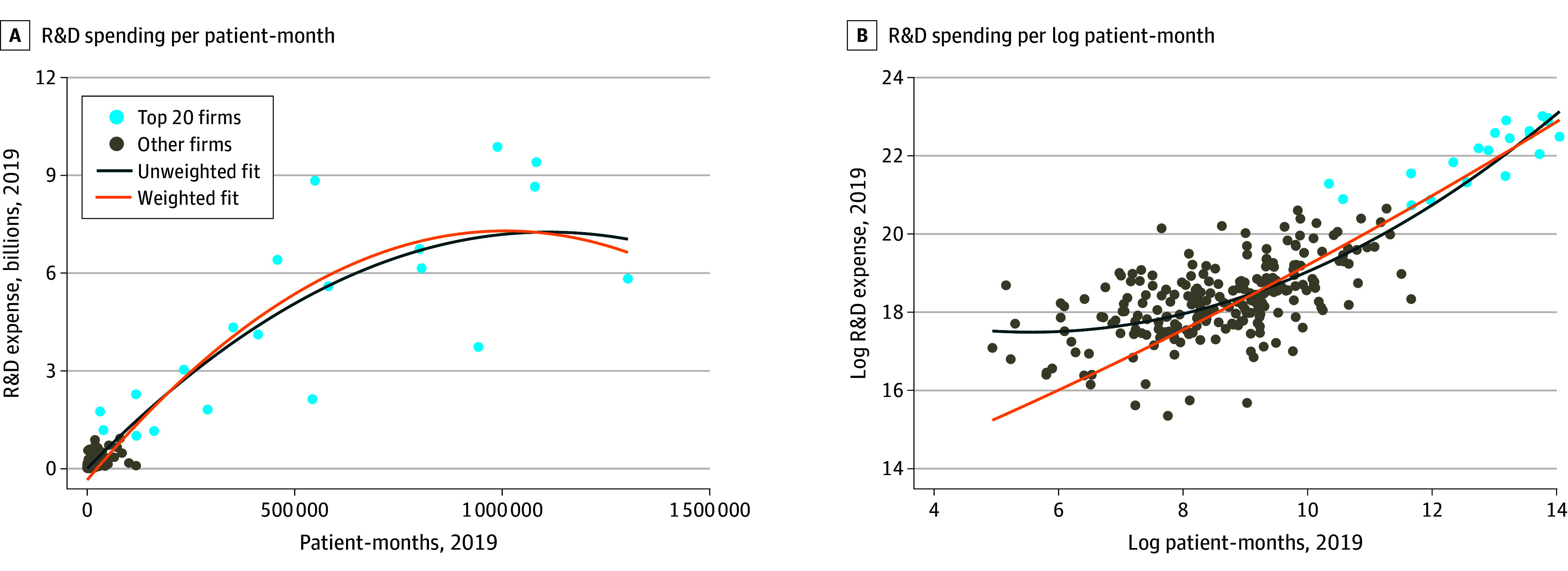
Research and Development (R&D) Spending Per Patient-Month in 2019 Figures include data from 248 companies with 2019 R&D expenses more than $0 and with more than 100 allocated patient-months in 2019.

Figure 1 plots the associations between R&D expenses and trial patient-months which form the basis for our main regression estimates. We compared 2019 R&D expense vs 2019 patient-months in levels with 2 quadratic best-fit trends, 1 weighted by patient-months and the other unweighted (Figure 1A). Then we calculated the same association with log-transformed R&D expense and patient-months to address the right-skew in both variables (Figure 1B). While the 20 largest firms remained outliers, the association between patient-months and R&D spending is generally linear, albeit with decreasing variance as log patient-months increased.

### Estimating the Association Between R&D Costs and Patient-Months

In our main regression, we estimated coefficients on log patient-months (ie, elasticities) of 0.92 and 0.62 for weighted and unweighted models, respectively (*P* < .001 for both; weighted *r*^2^ = 0.85; unweighted: r^2^ = 0.63). In other words, for every 1% increase in patient-months, we found an associated 0.92% increase in R&D expense. The magnitude of estimated costs per patient-month varied when estimating models on a stable panel firms contributing data in all 6 years, for the top 20 firms by R&D expenditures, for all firm-years outside the top 20 firms, and for US-based firms filing only 10-K (vs 20-F) annual reports to the SEC (eAppendix 2 in Supplement 1). However, there were 3 consistent findings. First, we found a strong association between log patient-months and log R&D expenditure, with coefficients ranging from 0.69 (the largest 20 firms) to 0.95 (stable panel of firms; *P* < .001 in all analyses). Second, while firm-level fixed R&D costs were consistently significant, their magnitudes were small compared with total firm-year R&D expenses (<$500 000). Third, we found little variation in inflation-adjusted R&D costs over time. eAppendix 2 in Supplement 1 presents results from other model specifications, including those including separate terms for oncology and other patient-months.

These results demonstrate a significant association between R&D costs and total patient-months. They also suggest that variable costs captured by patient-months comprise most total firm-level R&D spending, rather than firm fixed costs (which would not be captured by patient-months). Together, these 2 findings suggest that patient-months are a valid unit by which to measure R&D activity.

**Figure 2.**
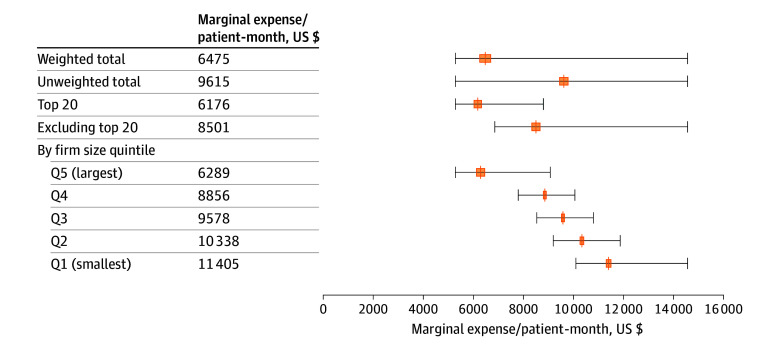
Estimated Marginal Research and Development Cost Per Patient-Month Lines indicate the mean estimated expense per patient-month adjusting for correlation within firm over time; boxes, 95% CIs; whiskers, range of marginal effects across firm-quarter-years. All results except the unweighted total are weighted by firm-year patient-months.

Figure 2 illustrates the distribution of the marginal increase in R&D spending from an additional patient-month for each firm-year in our data. The mean (SD) incremental cost per patient-month was $6475 ($1014) when weighting by firm-level patient-months and $9615 ($1662) without weighting (Figure 2). Estimated costs remained relatively constant over time but varied considerably by firm size. Mean (SD) weighted costs were $6176 ($562) for the top 20 firms by R&D spending and $8501 ($980) (37.6% higher) for smaller firms. Split differently, mean (SD) costs were $6289 ($713) in the largest fifth of firms vs $11 405 ($833) (81% higher) in the smallest fifth of firms, providing additional evidence of economies of scale in larger R&D operations at the firm or trial level.

### Estimated R&D Costs Per New Drug

**Figure 3.**
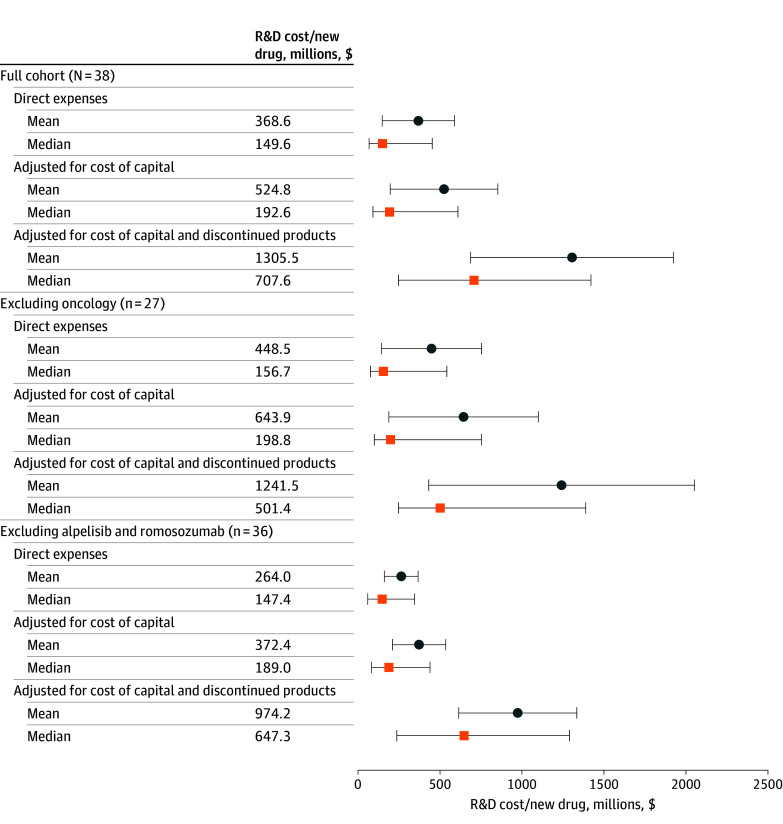
Estimated Research and Development (R&D) Costs Per New Drug Approved in 2019 (Millions) Blue circles (and whiskers) indicate the mean (with 95% CI) total estimated R&D cost for the relevant cohort of new drugs approved by the US Food and Drug Administration in 2019. Orange squares (and whiskers) indicate the median cost (and IQR).

We found highly variable and skewed costs per new drug approval after aggregating estimated R&D costs per patient-month across clinical trials for a cohort of 38 new drugs approved by the FDA in 2019 (Figure 3; eAppendix 4 in Supplement 1). For these drugs, the median (IQR) total R&D expense, reflecting all clinical trials and including adjustments for inflation, capitalization, and discontinued products, was approximately half of the mean expense, at $708 million ($247 million to $1.42 billion) vs mean (SD) $1.31 ($1.92) billion. Adjustments for discontinued products and to a lesser extent for the cost of capital led to substantially higher R&D costs: across all drugs, the median (IQR) direct R&D costs were $150 ($67.6-$453) million, compared with $193 ($91.5-$610) million adjusting for cost of capital and $708 million adjusting for both cost of capital and discontinued products (with correspondingly increasing mean [SD] costs of $369 [$684] million vs $525 million [$1.02 billion]; and $1.31 billion, respectively).

Excluding oncology drugs only modestly changed estimated means and medians, with the exception of lower median costs adjusting for discontinued products, given the relatively lower transition probabilities for oncology vs other drugs (Figure 3). Excluding just 2 drugs—1 oncology drug studied in many indications in parallel prior to approval (alpelisib) and an osteoporosis drug with uncommonly high clinical trial enrollment (romosozumab)—decreased mean adjusted R&D costs by one-quarter ($1.31 billion to $950 million, vs a <10% decrease for the median).

**Figure 4.**
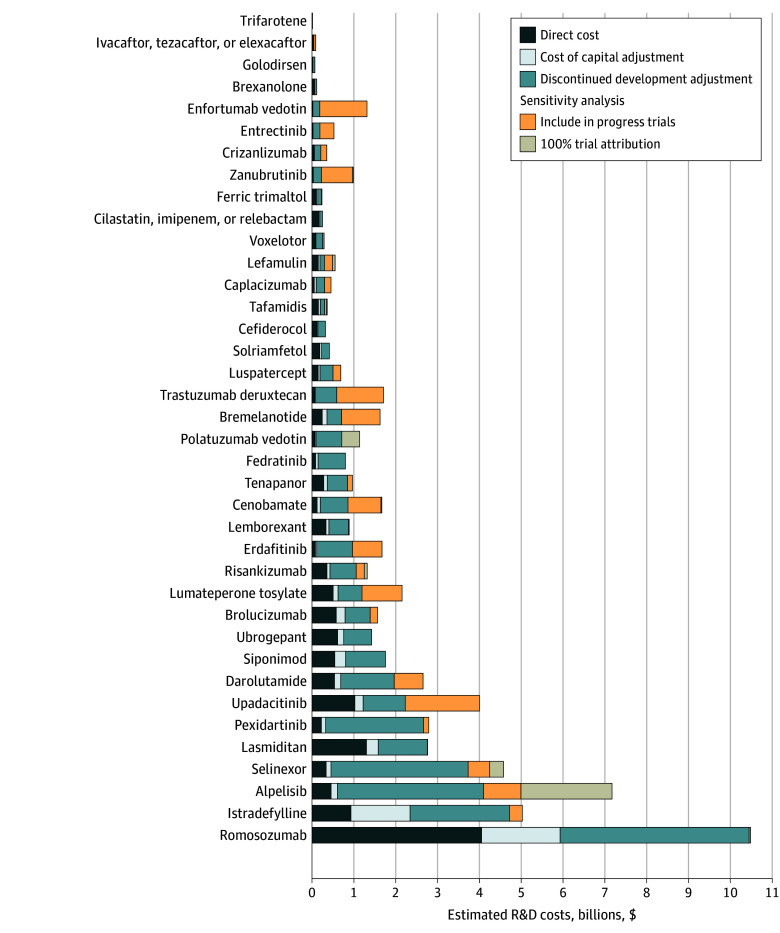
Estimated Research and Development (R&D) Costs Per New Drug Approved in 2019 Tabular, drug-level results are provided in eAppendix 4 in Supplement 1.

Figure 4 decomposes estimated R&D costs for each of the 38 new drugs into direct cost, cost of capital, and discontinued product adjustment components and adds 2 additional components excluded from the main results due to concerns they would unreasonably inflate cost estimates: one for in-progress trials begun prior to but completed after FDA approval, and the other attributing 100% of the cost of trials testing multiple drugs and with multiple sponsors to the new drug itself. Including both these costs where applicable as an upper bound estimate increased the median (IQR) cost of developing a new drug to $1.06 billion ($361 million to $1.75 billion) and mean (SD) cost to $1.72 ($2.13) billion. Separately, when using lower (6%) and higher (11%) cost of capital assumptions compared with 8.1% in our main approach, we found that the mean R&D cost per new drug ranged from $1.18 to $1.51 billion (compared with $1.31 billion at 8.1%) and that median costs per new drug ranged from $642 to $809 million (compared with $708 million at 8.1%) (eAppendix 5 in Supplement 1).

## Discussion

In this economic evaluation using a novel approach and after cost of capital and discontinued product adjustments, we estimated a median R&D cost of $708 million across a cohort of 38 drugs, with a considerably higher mean cost ($1.31 billion) driven by a small number of high-cost outliers. Mean R&D costs were 26% lower when excluding just 2 drugs. Reliable R&D cost estimates are essential to assessing the appropriateness of incentives for innovation (like government-granted monopolies from patents and regulatory exclusivity) and returns to drug developers. Given these highly skewed costs per new drug, median, rather than often-used mean, expenses may better reflect the typical case for the purposes of policy discussion.

Our work makes a methodological contribution in demonstrating a new way to estimate R&D costs per new drug. Our approach allocates all of firms’ R&D costs reported annually to the SEC, including early research, fixed, overhead, and marketing-focused R&D costs often omitted or imputed by assumption in other studies, onto a similarly broad firm-level total of annual patient-months. Cost per patient-month estimates serve as a flexible building block with application to other drug cohorts and in approximating R&D costs for government-sponsored development programs. While encompassing a wider set of R&D costs compared with most other studies, our mean and median per-drug R&D costs are within the range of other studies published over the past 20 years (with means from $539 million to $2.8 billion).^16,17^

Our study helps scope but does not directly address difficult distinctions between investments in R&D needed to bring a new drug to market, expand regulatory approval to additional patient populations and indications, and support marketing claims, lifecycle management, and other industry objectives. While the FDA’s Data Analysis and Search Host database (eg, as used by Sertkaya et al^20^) enumerates trials used by the agency to support regulatory approvals, these data are not publicly available. We ultimately opted for an inclusive approach in building our estimates by incorporating clinical trials for additional indications beyond than those initially (or ever) approved by the FDA, for academic collaborations, and for trials completed late into regulatory review. We included an even broader scope of trials and costs in sensitivity analyses. For a given drug, parallel development programs for multiple indications and populations are common, often with an initial, narrower indication qualifying for Orphan Drug Act tax and exclusivity benefits. Further investigation and policy discussion focusing on incentives for R&D investments to support early discovery and development by privately held firms, initial regulatory approval, broadening indications, and other purposes (eg, to support marketing claims) are crucial.

### Limitations

As with prior studies, there are important limitations of our data, methods, and assumptions. While our sample is large compared with prior studies, it excludes privately held companies and some larger drug companies not traded in the US (eg, Roche). Private, often venture capital–funded companies account for an increasing share of early-stage development,^31^ meaning our results may not be representative of total industry R&D costs. However, compared with 2019 global R&D spending, our sample covers approximately two-thirds of all industry spending; closely approximates spending by the top 10 largest firms, 9 of which are in our sample^32^; and includes many small companies and those without currently marketed products.

Our sample composition also changed over time, with smaller firms less likely to be included in earlier years. Given that most patient-months in our sample were captured by the top firms by R&D spending, any bias would likely not substantively affect our results, particularly when weighted. Nevertheless, we conducted a robustness check using a stable panel of firms.

We relied on companies’ reported differentiation between R&D expenses and costs of in-process R&D for acquired or in-licensed products. Acquisition and licensing expenses reflect both in-process development and assumptions on the future returns from R&D investments, which are difficult to disentangle empirically. Estimated R&D costs are not net of government subsidies, tax credits, or other incentives.

Other limitations relate to the Trialtrove data and our patient-month measure of R&D activity. First, some trials may be missing from the database, biasing estimates of R&D expense per patient-month upward. However, in comparisons between the scope of clinical trials included in Trialtrove and FDA review documents for a subset of 20 drugs, Trialtrove had as many or more trials listed in each case (eAppendix 6 in Supplement 1). Second, our patient-month measure uniformly spreads costs for patients across the entire study duration, without adjusting for changing enrollment or intensity of treatment over time. We caution against comparing estimated costs per patient-month to costs per month of active treatment. We aggregated patient-months by phase and for oncology trials separately in robustness checks.

Additionally, when estimating R&D expenses per new drug, we included all trials completed prior to the drug’s initial FDA approval date, including those for indications not covered by the approval. As a sensitivity analysis, we estimated costs including in progress trials that were ongoing at the time of FDA approval. Furthermore, we excluded phase 4 clinical trials, which the FDA sometimes requires as a condition of approval, as prior studies found only a fraction of these trials are actually completed.^33^

## Conclusions

This economic evaluation found a median (IQR) R&D cost of $708 million ($247 million to $1.42 billion) and a mean (SD) R&D cost of $1.31 billion ($150 million) across 38 new drugs approved by the FDA in 2019, after cost of capital and development program discontinuation adjustments, with substantially lower unadjusted direct costs (mean, $369 million; median, $150 million). Policymakers and researchers should consider median R&D costs and other nonparametric descriptive statistics describing the distribution of drug-level R&D costs alongside the more often used means when considering the typical R&D costs per new drug.

## Notes

Supplement 1. **eAppendix 1. **Additional Detail on Data and Main Methods **eAppendix 2.** Supplemental Results **eAppendix 3.** Cohort of New Molecular Entities Approved in 2019 **eAppendix 4.** Drug-Level Cost Estimates **eAppendix 5.** Alternative Cost of Capital Assumptions **eAppendix 6.** Comparison of Trialtrove R&D Activity for 2019 New Drugs to Trials in FDA Review Documents

Supplement 2.
Data Sharing Statement

## References

[1] Centers for Medicare & Medicaid Services. Medicare drug price negotiation. Updated October 22, 2024. Accessed June 24, 2024. https://www.cms.gov/inflation-reduction-act-and-medicare/medicare-drug-price-negotiation

[2] Pharmaceutical Research and Manufacturers of America. PhRMA statement on President Biden’s remarks to lower prescription drug prices. News release. August 12, 2021. Accessed October 15, 2021. https://phrma.org/resource-center/Topics/Cost-and-Value/PhRMA-Statement-on-President-Bidens-Remarks-to-Lower-Prescription-Drug-Prices

[3] Dubois P, De Mouzon O, Scott-Morton F, Seabright P. Market size and pharmaceutical innovation. RAND J Econ. 2015;46(4):844-871. doi:10.1111/1756-2171.12113

[4] Shaikh M, Del Giudice P, Kourouklis D. Revisiting the relationship between price regulation and pharmaceutical R&D investment. Appl Health Econ Health Policy. 2021;19(2):217-229. doi:10.1007/s40258-020-00601-9 32666383 PMC7902591

[5] CBO. Estimated Budgetary Effects of Public Law 117-169, to Provide for Reconciliation Pursuant to Title II of S. Con. Res. 14. Accessed June 24, 2024. https://www.cbo.gov/publication/58455

[6] Goldman DP, Joyce GF, Zheng Y. Prescription drug cost sharing: associations with medication and medical utilization and spending and health. JAMA. 2007;298(1):61-69. doi:10.1001/jama.298.1.61 17609491 PMC6375697

[7] Kirzinger A, Muñana C, Fehr R, Rousseau D; Kaiser Family Foundation. US public’s perspective on prescription drug costs. JAMA. 2019;322(15):1440. doi:10.1001/jama.2019.15547 31613335

[8] Conti R, Frank R, Gruber J. Addressing the trade-off between lower drug prices and incentives for pharmaceutical innovation. *Brookings*. November 15, 2021. Accessed February 8, 2022. https://www.brookings.edu/articles/addressing-the-trade-off-between-lower-drug-prices-and-incentives-for-pharmaceutical-innovation/

[9] Kesselheim AS, Avorn J, Sarpatwari A. The high cost of prescription drugs in the United States: origins and prospects for reform. JAMA. 2016;316(8):858-871. doi:10.1001/jama.2016.11237 27552619

[10] Keyhani S, Wang S, Hebert P, Carpenter D, Anderson G. US pharmaceutical innovation in an international context. Am J Public Health. 2010;100(6):1075-1080. doi:10.2105/AJPH.2009.178491 20403883 PMC2866602

[11] Lakdawalla D, Sood N. Incentives to Innovate. In: Danzon PM, Nicholson S, eds. The Oxford Handbook of the Economics of the Biopharmaceutical Industry. Oxford University Press; 2012.

[12] Arrow K. Economic Welfare and the Allocation of Resources for Invention. In: National Bureau Committee for Economic Research & Committee on Economic Growth of the Social Science Research Council, ed. The Rate and Direction of Inventive Activity: Economic and Social Factors. National Bureau of Economic Research; 1962:609-626.

[13] Nordhaus WD. The optimal life of a patent. In: Cowles Foundation, ed. Cowles Foundation Discussion Papers. Yale University; 1967:241.

[14] Winter SG. The logic of appropriability: from Schumpeter to Arrow to Teece. Res Policy. 2006;35(8):1100-1106. doi:10.1016/j.respol.2006.09.010

[15] Mulcahy AW, Schwam D, Lovejoy SL. International prescription drug price comparisons: estimates using 2022 data. *RAND Corporation*. February 1, 2024. Accessed November 21, 2024. https://www.rand.org/pubs/research_reports/RRA788-3.html 10.7249/rhq-11-03-05 PMC11147645 38855386

[16] Perehudoff K, Mara K, Hoen E. ’. What is the evidence on legal measures to improve the transparency of markets for medicines, vaccines and other health products: World Health Assembly resolution WHA72.8. Accessed November 21, 2024. https://iris.who.int/handle/10665/342474 34351727

[17] DiMasi JA, Grabowski HG, Hansen RW. Innovation in the pharmaceutical industry: new estimates of R&D costs. J Health Econ. 2016;47:20-33. doi:10.1016/j.jhealeco.2016.01.012 26928437

[18] Jayasundara K, Hollis A, Krahn M, Mamdani M, Hoch JS, Grootendorst P. Estimating the clinical cost of drug development for orphan versus non-orphan drugs. Orphanet J Rare Dis. 2019;14(1):12. doi:10.1186/s13023-018-0990-4 30630499 PMC6327525

[19] Rennane S, Baker L, Mulcahy A. Estimating the cost of industry investment in drug research and development: a review of methods and results. Inquiry. Published online February 16, 2022. doi:10.1177/00469580211059731 35170336 PMC8855407

[20] Sertkaya A, Beleche T, Jessup A, Sommers BD. Costs of drug development and research and development intensity in the US, 2000-2018. JAMA Netw Open. 2024;7(6):e2415445. doi:10.1001/jamanetworkopen.2024.15445 38941099 PMC11214120

[21] Prasad V, Mailankody S. Research and development spending to bring a single cancer drug to market and revenues after approval. JAMA Intern Med. 2017;177(11):1569-1575. doi:10.1001/jamainternmed.2017.3601 28892524 PMC5710275

[22] Adams CP, Brantner VV. Spending on new drug development. Health Econ. 2010;19(2):130-141. doi:10.1002/hec.1454 19247981

[23] Avorn J. The $2.6 billion pill—methodologic and policy considerations. N Engl J Med. 2015;372(20):1877-1879. doi:10.1056/NEJMp1500848 25970049

[24] Wouters OJ, Kesselheim AS. Quantifying research and development expenditures in the drug industry. JAMA Netw Open. 2024;7(6):e2415407. doi:10.1001/jamanetworkopen.2024.15407 38941103

[25] Husereau D, Drummond M, Augustovski F, ; CHEERS 2022 ISPOR Good Research Practices Task Force. Consolidated Health Economic Evaluation Reporting Standards 2022 (CHEERS 2022) statement: updated reporting guidance for health economic evaluations. Value Health. 2022;25(1):3-9. doi:10.1016/j.jval.2021.11.1351 35031096

[26] S&P Global. Seek & Prosper. Accessed November 21, 2024. https://www.spglobal.com/market-intelligence/en/campaigns/discover-capiqpro

[27] US Securities and Exchange Commission. Search Filings. Updated September 27, 2021. Accessed October 15, 2021. https://www.sec.gov/search-filings

[28] Citeline. Trialtrove. Accessed October 15, 2021. https://www.citeline.com/en/products-services/clinical/trialtrove

[29] Adams C, Herrnstadt H. CBO’s model of drug price negotiations under the Elijah E. Cummings Lower Drug Costs Now Act. Accessed October 15, 2021. https://www.cbo.gov/publication/56905

[30] Wong CH, Siah KW, Lo AW. Estimation of clinical trial success rates and related parameters. Biostatistics. 2019;20(2):273-286. doi:10.1093/biostatistics/kxx069 29394327 PMC6409418

[31] Teconomy Partners. Strengthening biopharmaceutical innovation: the growing role of corporate venture capital. Accessed October 15, 2021. https://phrma.org/-/media/Project/PhRMA/PhRMA-Org/PhRMA-Org/PDF/P-R/PhRMA-CVC-Report.pdf

[32] World Preview: Outlook to 2024. 12th ed. EvaluatePharma; 2019.

[33] Wallach JD, Luxkaranayagam AT, Dhruva SS, Miller JE, Ross JS. Postmarketing commitments for novel drugs and biologics approved by the US Food and Drug Administration: a cross-sectional analysis. BMC Med. 2019;17(1):117. doi:10.1186/s12916-019-1344-3 31203816 PMC6572730

