# Supplementary material for: Use of Clinical Trial Characteristics to Estimate Costs of New Drug Development
Source: JAMA Netw Open. 2025 Jan 6;8(1):e2453275. doi: 10.1001/jamanetworkopen.2024.53275 (PMC11704977; doi:10.1001/jamanetworkopen.2024.53275)
Supplement: Supplement 1. — eAppendix 1. Additional Detail on Data and Main Methods eAppendix 2. Supplemental Results eAppendix 3. Cohort of New Molecular Entities Approved in 2019 eAppendix 4. Drug-Level Cost Estimates eAppendix 5. Alternative Cost of Capital Assumptions eAppendix 6. Comparison of Trialtrove R&D Activity for 2019 New Drugs to Trials in FDA Review Documents [file jamanetwopen-e2453275-s001.pdf]

Supplemental Online Content

Mulcahy A, Rennane S, Schwam D, Dickerson R, Baker L, Shetty K. Use of clinical trial characteristics to estimate costs of new drug development. *JAMA Netw Open*. 2025;8(1):e2453275. doi:10.1001/jamanetworkopen.2024.53275

eAppendix 1. Additional Detail on Data and Main Methods
eAppendix 2. Supplemental Results
eAppendix 3. Cohort of New Molecular Entities Approved in 2019
eAppendix 4. Drug-Level Cost Estimates
eAppendix 5. Alternative Cost of Capital Assumptions
eAppendix 6. Comparison of Trialtrove R&D Activity for 2019 New Drugs to Trials in FDA Review Documents

This supplemental material has been provided by the authors to give readers additional information about their work.

© 2025 Mulcahy A et al. *JAMA Network Open.*

eAppendix 1. Additional Detail on Data and Main Methods

1a. Firm selection details

We identified pharmaceutical companies publicly traded on the New York Stock Exchange, NASDAQ, or AMEX with Standard Industrial Classification codes 2834, 2835, 2836 in the S&P database (a total of 681 companies) and pulled information on reported assets for each company from their annual FY 2018 SEC filing.[1] We excluded companies producing only generic drugs, veterinary products, or technology platforms related to drug development after our review of SEC filings. Then, we ranked the remaining companies in descending order by assets and selected the top 200 to be included in our sample.

Our goal in ranking companies by assets was to capture the largest pharmaceutical firms with novel drug R&D activity. However, there are several methods that could be used to measure firm size. Dang, Li, and Yang (2018) study three of the most common measures of firm size: total assets, market capitalization, and total sales and find that these three measures yield consistent findings in corporate finance models of R&D.[2] Because some pharmaceutical companies with modest assets may still be engaged in substantial R&D activity, we included 68 additional companies ranking in the top 200 pharmaceutical companies based on market cap as reported in the S&P database that were not included in the top 200 firms by reported assets. Our final sample included 268 firms defined by SEC Central Index Key (CIK) that were publicly traded in the U.S. for at least one year between 2014-2019. Compared to 2019 estimates of global R&D expenditures from Evaluate Pharma, we estimate that our final sample consistently covers approximately two-thirds of all pharmaceutical R&D spending globally between 2014

[1] 2018 data were the most recent data when we established our sample of firms. We subsequently updated our SEC abstraction to include 2019 data.

[2] Dang, C., Z.F. Li, and C. Yang, *Measuring firm size in empirical corporate finance. J of Banking Finance*, 2018. **86**: p. 159-176.

© 2025 Mulcahy A et al. *JAMA Network Open.*

and 2019, and very closely approximates spending by the top 10 largest firms, 9 of which are included in our sample.[3]

1b. Firm-level R&D spending adjustments

We converted annual R&D expenses to 2019 dollars using the "Pharmaceuticals for human use, prescription" annual average producer price index from the U.S. Bureau of Labor Statistics. A total of 32 firms (16%) filed a 20-F in at least one year. In some cases, amounts in 20-F filings were reported in foreign currency. We converted these to U.S. dollars at average annual exchange rates.[4] For firms using a fiscal year starting on a date other than January 1, we weighted data proportionally across fiscal years to align with calendar years (this required abstracting 2020 SEC data for some firms). Some conglomerate firms (e.g., Johnson & Johnson) reported separate R&D expenses for different business units. We collected pharmaceutical R&D expenses specifically in these cases.

1c. Trialtrove overview

The next sections describe a series of sequential steps to prepare Trialtrove data for analysis. As a preview, we first matched firm names from SEC with Trialtrove sponsor names. Second, we extracted all trials that were active at some point between 2014-2019 with a selected firm as a sponsor. Third, we imputed missing trial durations and enrollment as necessary using a hot-deck imputation approach. Using these trial inputs, we calculated patient-months for each trial. We divided a trial's total patient-months evenly across sponsors in cases where there was

[3] *World Preview 2019, Outlook to 2024*, Evaluate Pharma. 2019.
[4] IRS. *Yearly Average Currency Exchange Rates*. 2021 June 26, 2021 [cited October 15, 2021]; Available from: https://www.irs.gov/individuals/international-taxpayers/yearly-average-currency-exchange-rates

© 2025 Mulcahy A et al. *JAMA Network Open.*

more than one sponsor and made several adjustments to account for mergers and acquisitions during our study period.

1d. Matching firms and extracting trials in Trialtrove

The only common unique identifier for companies to match the SEC filings and Trialtrove is company name. We took a broad approach to identify the correct company names in Trialtrove. We observed several company name inconsistencies in the Trialtrove data. For instance, Teva Pharmaceuticals is referred to as "Teva" and "Teva Pharmaceutical Industries, Ltd". In order to allow for the maximum number of company names, we used a set of truncated search strings (i.e., "Teva Pharmaceutical Industries Ltd." was truncated to "Teva"). If the truncated search string was not sufficiently specific (e.g., "Ra" for "Ra Pharmaceuticals"), we included the full name of the company. We utilized the Trialtrove "contains" function instead of the Trialtrove "is" function to identify the broadest set of possible trials and then manually checked each sponsor.

There were 1,037 unique sponsor strings (excluding co-sponsors) that were identified using the truncated "contains" search methodology. Two researchers reviewed this list and identified inconsistencies for *ad hoc* correction. For instance, the search term "biogen" resulted in matches for Biogen (correctly identified), Biogene Pharmaceuticals (incorrectly identified), and Biogenomics (incorrectly identified). There were 955 unique strings after excluding extraneous sponsors. These unique strings separately account for each combination of sponsors involved in a trial. For example, "Pfizer and BioNTech" and "Pfizer" would appear as separate sponsor strings.

After identifying all unique sponsor strings that included one of the companies in our sample, we extracted all trials associated with these names in the Trialtrove database. We

excluded trials that ended, closed or were terminated before the beginning of our study period in 2014; trials without any status updates after 2013 but were not explicitly listed as ended or terminated; trials where both start and end dates were missing; trials that are listed as "planned, but never initiated" in Trialtrove, and trials indicated as planned with start dates after the end of our study period in 2019.

1e. Imputing missing data

To calculate patient-month measures, we require data from Trialtrove on patient enrollment (i.e., accrual), and trial duration. In cases where one or both of these inputs is missing, we imputed the values from observed cases.

eTable 1: Average share of trials missing key inputs, by trial phase

Trial Phase	Patient accrual	Trial Duration
I	13%	25%
II	10%	10%
III	11%	10%
IV	10%	18%
Average	11%	15%

Source: Author analysis of 2014-2019 Trialtrove R&D input data.

Approximately 11 percent of trials were missing data on accrual and 15 percent are missing data on the start or end date of the trial (which we use to measure trial duration; eTable 1). Missing data was more common in earlier phase trials.

We implemented a multiple imputation approach to address these missing inputs. For each trial missing one or more inputs, we randomly selected one trial with complete information in the same phase and therapeutic area and assigned the missing values for the trial with missing inputs to be equal to the values in the randomly selected trial. After all missing inputs were

imputed, we calculated our main independent variable (patient- months) for the full database, including imputed values. We repeated this exercise five times.

eFigure 1 shows the median values for patient accrual and trial duration based only on the cases where we observe complete data, and for each of our five imputations. The medians of the observed and imputed distributions are very similar. For our analyses, we used the average of the resulting estimates for accrual and trial duration across the five imputations.

eFigure 1: Median values of patient accrual and trial duration, observed and imputed values

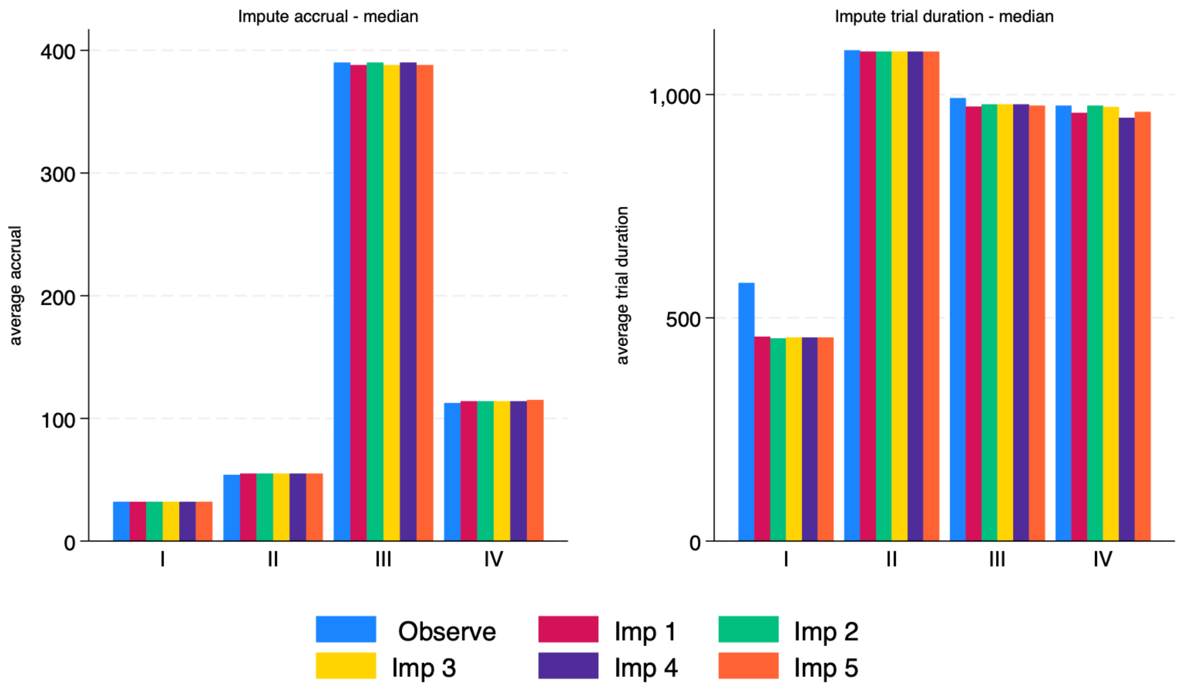

Source: Author analysis of 2014-2019 Trialtrove R&D input data.

1f. Allocating patient-months across sponsors and trial years

© 2025 Mulcahy A et al. *JAMA Network Open.*

A significant share of trials in our Trialtrove analytic file, 41.9% of trials, had multiple sponsors. Of these trials, 70.3% had 2 sponsors, 19.6% had 3 sponsors, and 10.1% had 4 or more sponsors. There were many different sponsor combinations. For the co-sponsors, 49.60% were academic institutions, 18.10% were industry, 10.57% were government organization, 2.12% were non-profit organizations, and 19.61% were other types of co-sponsors.

We allocated activity equally among all listed trial sponsors. For our analysis, however, we excluded the share of trial activity allocated to sponsors that are not included in our sample. For example, if there was a trial with two sponsors and one of them was not in our sample, we only included 50% of trial activity in the database – attributed to the sponsor included in our analysis.

We also investigated the type of co-sponsor. Of the 41.87% of trials with multiple sponsors, 39.1% had only academic co-sponsors, 13.8% had only industry co-sponsors, 3.1% had only industry and academic co-sponsors, and 44% had another co-sponsor combination. The high share of academic co-sponsors raises some questions about the extent of involvement of the industry partner. For example, the industry partner may be actively involved in the R&D work, or may simply provide study drug product, in which case the R&D contribution and expenses incurred may be minimal. As a result, we conduct a robustness check excluding all trials with an industry partner and only one academic partner.

After allocating total trial activity across sponsors, we uniformly spread trial activity across the full duration of the trial, mapping the trial start and stop dates to months while allowing for fractional months. However, we also create an indicator for the year in which the trial began, to capture the fact that there may be fixed start-up costs at the beginning of a trial. We include this variable in a robustness check.

© 2025 Mulcahy A et al. *JAMA Network Open.*

1g. Adjustments for mergers and acquisitions

Next, we allocate trial activity across trials where we observed merger and acquisition (M&A) activity. There are 5 specific types of possible M&A activity that we investigated:

The first was if there was no M&A involved. In this scenario, we allocated all the trial to the trial sponsor.

The second scenario was if an out-of-scope co-sponsor was involved with M&A activity. Because this trial activity is not counted in our final data set, we left this as is and did not adjust the allocation.

The third scenario was if there was M&A involved with one of the within scope companies. We searched for the dates of the merger or acquisition by hand if the trial sponsor was involved with 50 or more trials. This covered 61% of all trials that involved M&A activity. For these trials, we assumed uniform trial cost across the full length of the trial and allocated the trial proportionally based upon the date when the acquisition or merger occurred relative to the total trial duration. After researching these dates, the most common scenario was that the M&A activity took place before 2014, so the entirety of the trial activities was allocated to the acquiring firm. However, we did observe several M&A events that took place during our analysis period.

For the remaining 39% of trials with M&A activity, we assumed that the acquisition took place at the midpoint of the trial and allocated the second half of the trial to the acquiring firm. Furthermore, we assumed that only the two most recent firms were involved with the trial. However, some trials/firms have extensive M&A histories.

The fourth scenario was if there was two or more of the within scope companies involved (i.e. one within scope company acquiring another within scope company or one within scope

company acquiring the subsidiary of another within scope company). We investigated these cases by manually and switched the ownership of the trial on the acquisition date.

The final scenario was if there was a joint venture. We split the allocation of the trial equally amongst each company in the joint venture. For a joint venture with 3 companies, each company would be allocated 1/3 of the trial.

© 2025 Mulcahy A et al. *JAMA Network Open.*

eAppendix 2. Supplemental Results

2a. Descriptive statistics at different exclusion thresholds

eTable 2 presents descriptive statistics on the number of firms, mean firm-year R&D expense, and mean firm-year patient-months for three groups of firms: (1) firms with any R&D expense and activity; (2) firms with any R&D expense and at least 100 patient-months (as in our main results; and (3) firms with any R&D expense and at least 200 patient-months. We present results below using data from all firms with R&D expense and with the more stringent 200 patient-month threshold as a sensitivity analysis around our main result.

eTable 2: Additional firm sample and firm-year descriptive statistics

	**Number of firms (% of 268 total firms)**						
	All Yrs.	*2014*	*2015*	*2016*	*2017*	*2018*	*2019*
With both R&D expense and activity	268 (100%)	186 (69.4%)	210 (78.4%)	229 (85.4%)	240 (89.6%)	257 (95.9%)	256 (95.5%)
Also with >100 patient-months	264 (98.5%)	172 (64.2%)	200 (74.6%)	215 (80.2%)	230 (85.8%)	246 (91.8%)	248 (92.5%)
Also with >200 patient-months	263 (98.1%)	167 (62.3%)	191 (71.3%)	209 (78.0%)	222 (82.8%)	240 (89.6%)	244 (91.0%)
	**Mean R&D expense ($mils; 95% CI)**						
	All Yrs.	*2014*	*2015*	*2016*	*2017*	*2018*	*2019*
With both R&D expense and activity	496.4 (417.6-575.3)	469.2 (262.6-675.9)	479.7 (287.5-671.9)	488.6 (296.6-680.7)	494.9 (304.8-685)	517.2 (324.8-709.7)	517.5 (331.6-703.5)
Also with >100 patient-months	520.3 (437.7-602.9)	505.8 (283.2-728.5)	502 (300.6-703.3)	518.7 (314.8-722.7)	515.2 (317.2-713.1)	539.1 (338.4-739.7)	532.7 (341-724.3)
Also with >200 patient-months	534.6 (449.6-619.6)	520.4 (291.4-749.3)	524.8 (314.5-735.2)	532.3 (322.8-741.9)	532.1 (327.4-736.9)	550.7 (345.2-756.2)	540.5 (345.8-735.1)
	**Mean patient-months (thousands; 95% CI)**						
	All Yrs.	*2014*	*2015*	*2016*	*2017*	*2018*	*2019*

© 2025 Mulcahy A et al. *JAMA Network Open.*

With both R&D expense and activity	26.0 (21.2-30.7)	33.7 (17.4-50.0)	30.3 (16.3-44.2)	26.6 (15.3-37.9)	23.5 (13.4-33.6)	21.1 (12.2-30.0)	23.4 (12.9-33.9)
Also with >50 patient-months	28.2 (23.0-33.3)	36.9 (19.1-546)	33.1 (17.9-48.3)	29.9 (17.3-42.5)	25.3 (14.4-36.1)	23.0 (13.4-32.7)	24.5 (13.5-35.4)

Source: Author analysis of 2014-2019 SEC R&D expense and Trialtrove data.
Notes: For the reported number of firms, "all years" reports the number of unique firms with R&D expenses and >100 patient-months across all six years. The total number of firm-years was 1,311. For mean R&D expense and patient months, the "all years" means are calculated across all firm-year records.

2b. Supplemental regression analysis results: Firm subgroups

eTable 3 reports estimated coefficients from firm subgroup robustness checks. Our main results use data from all firm-years with >100 patient-months. We estimated the same regression model for a subset of stable panel firms contributing data in all six years, for the top 20 firms by R&D expenditures, for all firm-years outside the top 20 firms, and for U.S.-based firms filing only 10-K (vs. 20-F) annual reports to the SEC.

In the last case, we investigated 10-K vs. 20-F subgroups due to differences in accounting practices: U.S.-based 10-K filers must use GAAP while 20-F filers have more flexibility to use International Accounting Standard Board's (IASB) International Financial Reporting Stands (IFRS) (see SEC, 2007 for the final ruling). There are some differences between GAAP and IFRS in how companies report research and development expenses. The primary difference in accounting for R&D under these two standards boils down to recognizing the economic value of R&D (Turnlington, Fafatas, and Goad Oliver, 2019). Under GAAP, all costs/expenses associating with developing a new product and bringing it to market fall under R&D and are expensed as incurred. Under IFRS, once a product has been demonstrated, the newly developed

© 2025 Mulcahy A et al. *JAMA Network Open.*

product can be recognized as an intangible asset on the company's balance sheet via a capitalized cost and are amortized over time.

© 2025 Mulcahy A et al. *JAMA Network Open.*

eTable 3: Estimated regression coefficients for subsets of firms

	(1)<br>All firm-years	(2)<br>Stable panel	(3)<br>Top 20	(4)<br>Outside top 20	(5)<br>10-K only
Log patient-months	0.915***<br>(0.0370)	0.947***<br>(0.0373)	0.685***<br>(0.0794)	0.845***<br>(0.0241)	0.946***<br>(0.0454)
Year=2015 (vs. 2014)	0.0170<br>(0.0826)	0.0780<br>(0.0619)	0.0462<br>(0.0551)	0.101<br>(0.121)	0.0543<br>(0.0498)
Year=2016	0.0842<br>(0.0883)	0.138*<br>(0.0739)	0.106<br>(0.0689)	0.0954<br>(0.127)	0.129<br>(0.0791)
Year=2017	0.0738<br>(0.104)	0.141<br>(0.0895)	0.0933<br>(0.0832)	0.0436<br>(0.132)	0.199*<br>(0.104)
Year=2018	0.167<br>(0.108)	0.256***<br>(0.0797)	0.184**<br>(0.0713)	0.131<br>(0.163)	0.284***<br>(0.103)
Year=2019	0.119<br>(0.0777)	0.178**<br>(0.0705)	0.0792<br>(0.0631)	0.197<br>(0.234)	0.228**<br>(0.0953)
Constant	9.906***<br>(0.479)	9.456***<br>(0.470)	13.13***<br>(1.060)	10.36***<br>(0.299)	9.528***<br>(0.578)
Observations	1,311	876	120	1,191	826
R-squared	0.851	0.853	0.653	0.833	0.839

Robust standard errors clustered at the firm level in parentheses
*** p<0.01, ** p<0.05, * p<0.1

Source: Author analysis of 2014-2019 SEC R&D expense and Trialtrove data.

2c. Supplemental regression analysis results: Trial restrictions

As described in the main body and in S1 above, we allocated patient-months in proportion to the number of sponsors and in other circumstances. As a robustness check, we estimated models under different allocation assumptions, including allocating trials with academic co-sponsors to the industry sponsor at 0% (i.e., excluding these patient-months). As noted above, many trials and especially earlier clinical trials had both industry and academic co-sponsors listed in TrialTrove and clinicaltrials.gov: we could not determine whether the industry partner involvement was limited to providing drug product or expanded to directly financing the trial. The weighted and unweighted average marginal effects of an additional patient-month were $7,794 and $10,121 after this change, respectively, compared to $6,475 and $9,615 in our main

© 2025 Mulcahy A et al. *JAMA Network Open.*

results. These higher costs per patient-month stems mechanically from shrinking the aggregated firm-year count of patient-months. Unfortunately, without additional information on drug companies' roles in these trials, we cannot more accurately allocate patient-months. Our main approach of splitting patient-months N-ways may be a reasonable compromise.

In another sensitivity analysis, we calculated average marginal effects excluding trials with a sponsor involved in M&A during the study period. We found only slight differences in weighted and unweighted average marginal effects of an additional patient-month after this exclusion: $6,420 and $9,477, respectively, compared to $6,475 and $9,615 in our main results.

2d. Supplemental regression analysis results: Model specifications

We also estimated regression models with additional covariates and replacing our aggregate log count of patient-months with more granular counts. eTable 4 reports estimated coefficients from these models. We added 1 to these narrower counts of patient counts before log transformation to address many more 0 values when tallying patient-months at more granular levels. While costs per patient-month should conceptually vary across therapeutic areas, these differences are in practice difficult to disentangle empirically because many drug developers – including the top 20 firms by patient-months – develop drugs across broad sets of therapeutic areas, and because clinical trial enrollment and duration can vary widely even within a therapeutic area (e.g., cardiovascular drugs). Based on exploratory analysis, we ultimately included separate patient-month counts for oncology and non-oncology trials in a robustness check rather than separate patient-month count terms for every therapeutic area.

As discussed in the main body, including patient-months by phase or separately for oncology and other trials decreases the magnitude of estimated elasticities, with an offsetting

© 2025 Mulcahy A et al. *JAMA Network Open.*

increase in the estimated constant term. These shifts in magnitude may be due to highly correlated log patient-months between phases.

Separately, we estimated random and fixed effects models controlling for time-invariant firm-level characteristics as an additional check and found that the coefficients sign and significance are similar, although the magnitude is smaller (results not reported). In our random effects model, we found a large share of the variance in R&D costs over time was due to firm-specific factors.

eTable 4: Estimated regression coefficients for alternative specifications

	(1)<br>Main results	(2)<br>Starts	(3)<br>By-phase	(4)<br>By-phase excl. P1	(5)<br>Oncology vs. other
Log patient-months	0.915***<br>(0.0370)	0.876***<br>(0.0441)			
New trial starts		0.00238<br>(0.00250)			
Log pt.-months, P1			0.167***<br>(0.0204)		
Log pt.-months, P2			0.164***<br>(0.0189)	0.249***<br>(0.0240)	
Log pt.-months, P3			0.199***<br>(0.0190)	0.230***<br>(0.0294)	
Log pt.-months, P4			0.100***<br>(0.0157)	0.136***<br>(0.0211)	
Log pt.-months, onc.					0.223***<br>(0.0456)
Log pt.-months, oth.					0.358***<br>(0.0394)
Year=2015 (vs. 2014)	0.0170<br>(0.0826)	0.0941<br>(0.0889)	0.0676<br>(0.0518)	0.0759<br>(0.0499)	0.0242<br>(0.0528)
Year=2016	0.0842<br>(0.0883)	0.256***<br>(0.0986)	0.132**<br>(0.0596)	0.156***<br>(0.0585)	0.123*<br>(0.0676)
Year=2017	0.0738<br>(0.104)	0.313***<br>(0.119)	0.163**<br>(0.0694)	0.200***<br>(0.0713)	0.178**<br>(0.0813)
Year=2018	0.167<br>(0.108)	0.463***<br>(0.128)	0.286***<br>(0.0806)	0.326***<br>(0.0789)	0.294***<br>(0.0878)
Year=2019	0.119<br>(0.0777)	0.453***<br>(0.0984)	0.276***<br>(0.0609)	0.305***<br>(0.0585)	0.340***<br>(0.0629)
Constant	9.906***	9.983***	14.86***	14.73***	14.82***

© 2025 Mulcahy A et al. *JAMA Network Open.*

	(1)<br>Main results	(2)<br>Starts	(3)<br>By-phase	(4)<br>By-phase excl. P1	(5)<br>Oncology vs. other
	(0.479)	(0.471)	(0.251)	(0.332)	(0.288)
Observations	1,311	1,311	1,311	1,311	1,311
R-squared	0.851	0.850	0.911	0.886	0.845

Robust standard errors clustered at the firm level in parentheses
*** p<0.01, ** p<0.05, * p<0.1

Source: Author analysis of 2014-2019 SEC R&D expense and Trialtrove data.

© 2025 Mulcahy A et al. *JAMA Network Open.*

eAppendix 3. Cohort of New Molecular Entities Approved in 2019

We constructed R&D cost estimates for 38 of 48 total new molecular entities approved by the FDA in 2019. We excluded 4 contrast agents and radiopharmaceuticals because drugs in these categories have atypical clinical development programs (Brilliant Blue G Ophthalmic Solution, air polymer-type A, fluorodopa F 18, Ga-68-DOTATOC); 2 drugs to treat tropical diseases without clinical trial activity in the United States (triclabendazole and pretomanid); and 4 other drugs for miscellaneous reasons, one a drug from a privately held company and no reported clinical trials (pitolisant), another an active ingredient qualifying as a new molecular entity but closely related to an existing marketed drug (prabotulinum toxin A), and two with some clinical trials in TrialTrove but without any allocated patient-months prior to approval (afamelanotide and givosiran).

© 2025 Mulcahy A et al. *JAMA Network Open.*

eAppendix 4. Drug-level Cost Estimates

eTable 5. Drug-level cost estimates illustrated in Figure 4

Note: Table sorted in descending order in terms of estimated costs with cost of capital and discontinued drug adjustments.

Generic name	Brand name	Sponsor	Ther. Category	Direct cost	Cost of capital adjustment	Discontinued development adjustment	Sensitivity analysis: Include in progress trials	Sensitivity analysis: 100% trial attribution
romosozumab	Evenity	Amgen	Metabolic	4,049.2	5,927.5	10,443.9	10,486.5	10,486.5
istradefylline	Nourianz	Kyowa Kirin	CNS	927.5	2,340.5	4,718.3	5,035.6	5,035.6
alpelisib	Piqray	Novartis	Oncology	452.6	609.5	4,095.5	5,001.5	7,176.7
selinexor	Xpovio	Karyopharm	Oncology	335.8	451.1	3,730.4	4,256.0	4,582.5
lasmiditan	Reyvow	Lilly	CNS	1,298.9	1,580.5	2,751.8	2,751.8	2,764.6
pexidartinib	Turalio	Daiichi	Oncology	219.9	322.1	2,668.0	2,798.4	2,798.4
upadacitinib	Rinvoq	AbbVie	Autoimmune	1,022.2	1,220.2	2,229.7	4,011.8	4,011.8
darolutamide	Nubeqa	Bayer	Oncology	534.6	682.5	1,962.2	2,662.3	2,662.3
siponimod	Mayzent	Novartis	CNS	541.7	803.1	1,752.0	1,752.0	1,752.0
ubrogepant	Ubrelvy	Allergan	CNS	611.0	754.2	1,420.9	1,420.9	1,420.9
brolucizumab	Beovu	Novartis	Ophthalmology	576.5	789.5	1,388.3	1,574.1	1,574.1
lumateperone tosylate	Caplyta	Intra-Cellular	CNS	502.7	619.6	1,192.2	2,161.1	2,161.1
risankizumab	Skyrizi	AbbVie	Autoimmune	354.3	427.2	1,054.9	1,258.0	1,325.4
erdafitinib	Balversa	J&J	Oncology	79.2	109.9	963.7	1,680.3	1,680.3
lemborexant	Dayvigo	Eisai	CNS	331.0	403.1	872.3	875.1	877.4
cenobamate	Xcopri	SK Life Science	CNS	119.9	198.8	858.1	1,655.6	1,659.5
tenapanor	Ibsrela	Ardelyx	Metabolic	277.4	366.1	848.5	972.3	972.3
fedratinib	Inrebic	Sanofi	Oncology	83.4	143.6	798.2	798.2	798.2
polatuzumab vedotin	Polivy	Roche	Oncology	67.6	92.8	708.1	708.1	1,144.7
bremelanotide	Vyleesi	AMAG	Genitourinary	240.9	355.3	707.2	1,634.4	1,634.4
trastuzumab deruxtecan	Enhertu	AstraZeneca	Oncology	69.5	75.8	587.5	1,714.8	1,714.8
luspatercept	Reblozyl	BMS	Metabolic	146.1	189.6	501.4	694.1	694.1

© 2025 Mulcahy A et al. *JAMA Network Open.*

Generic name	Brand name	Sponsor	Ther. Category	Direct cost	Cost of capital adjustment	Discontinued development adjustment	Sensitivity analysis: Include in progress trials	Sensitivity analysis: 100% trial attribution
solriamfetol	Sunosi	Jazz	CNS	185.6	225.4	412.7	412.7	412.7
cefiderocol	Fetroja	Shionogi	Infectious disease	116.5	139.2	319.6	319.6	319.6
tafamidis	Vyndaqel	Foldrx	Other	150.3	195.6	301.6	351.1	351.6
caplacizumab	Cablivi	Ablynx	Cardiovascular	51.0	99.6	299.0	461.5	461.5
lefamulin	Xenleta	Nabriva	Infectious disease	148.8	188.3	295.8	496.8	559.3
voxelotor	Oxbryta	Global Blood	Metabolic	77.0	91.5	254.2	295.5	295.5
cilastatin/imipenem/relebactam	Recarbrio	Merck	Infectious disease	156.7	183.0	246.6	246.6	246.6
ferric trimaltol	Accrufer	Shield	Metabolic	104.0	135.1	226.9	230.7	230.7
zanubrutinib	Brukinsa	BeiGene	Oncology	25.5	28.9	222.7	975.4	979.3
crizanlizumab	Adakveo	Novartis	Metabolic	37.0	55.0	208.2	361.1	361.1
entrectinib	Rozlytrek	Genentech	Oncology	16.0	22.9	176.7	532.6	532.6
enfortumab vedotin	Padcev	Astellas	Oncology	13.0	20.0	175.6	1,323.6	1,323.6
brexanolone	Zulresso	Sage	CNS	48.5	57.4	109.6	109.6	109.6
golodirsen	Vyondys 53	Sarepta	CNS	12.9	15.8	65.4	65.4	65.4
ivacaftor/tezacaftor/elexacaftor	Trikafta	Vertex	Autoimmune	20.6	20.2	29.5	96.7	96.7
trifarotene	Aklief	Galderma	Other	1.8	2.8	11.8	11.8	11.8

© 2025 Mulcahy A et al. *JAMA Network Open.*

eAppendix 5. Alternative Cost of Capital Assumptions

Adjustments for cost of capital account for the fact that firms must invest funds in development projects long before they reap the returns of their investment. If those funds had been invested elsewhere, they could have generated interest which would increase their value. The foregone opportunity of getting this investment return during the drug development period is known as the cost of capital. Because drug development lasts over several years, and investment values compound over time, the foregone returns can be substantial. Because one can never know exactly firms would have invested these funds if they had been used for development, it is unclear what the counterfactual gain would have been, and as a result what rate should be used to estimate the foregone returns. Existing work uses a variety of cost of capital rates to estimate this cost. Our primary estimates use an 8.1 percent cost of capital, the rate most recently used by the CBO.[5] In these sensitivity analyses, we present results for capitalized costs with several alternative rates, including 11 percent (an estimate commonly used in the literature[6]), as well as 6 percent for a low-range estimate.

To account for the cost of capital, we divide patient-months uniformly over the duration of drug development apply a compounded interest formula to the monthly cost using the chosen cost of capital rate. However, in reality, patient-months are likely weighted more heavily towards the end of clinical trials after site and patient enrollment are well underway. If that is the case, then capitalized trial-level costs would be lower, as a larger share of the incurred direct costs would be happening closer to the end of the trial and less compounding would occur. In other

[5] CBO, *Budgetary Effects of Public Law 117-169*. 2022.
[6] DiMasi, J.A., H.G. Grabowski, and R.W. Hansen, *Innovation in the pharmaceutical industry: New estimates of R&D costs.* J Health Econ, 2016. **47**: p. 20-33.
Sertkaya A, Beleche T, Jessup A, Sommers BD. Costs of Drug Development and Research and Development Intensity in the US, 2000-2018. *JAMA Netw Open.* 2024;7(6):e2415445.

© 2025 Mulcahy A et al. *JAMA Network Open.*

words, the estimates presented here (under the assumption that trial costs are uniformly distributed across the duration of the trial) may be over-estimates of capitalized costs.

eFigure 2 below shows the resulting range in estimates under varying cost of capital assumptions. In the top panel, we show the estimated range of capitalized costs, and the bottom panel shows the range once accounting for failures. The cost of capital used is shown on the y-axis (ranging from 6 to 11%) and the estimates for 8.1% match those shown in the paper. In total, varying the cost of capital results in varying mean costs from $475 to $605 million, and median costs from $177 to $212 million. Once accounting for failures, the range for the mean increases to $1.2 to $1.5 billion, and the range for the median increases to $642 to $809 million.

eFigure 2: R&D cost estimates under varying cost of capital assumptions

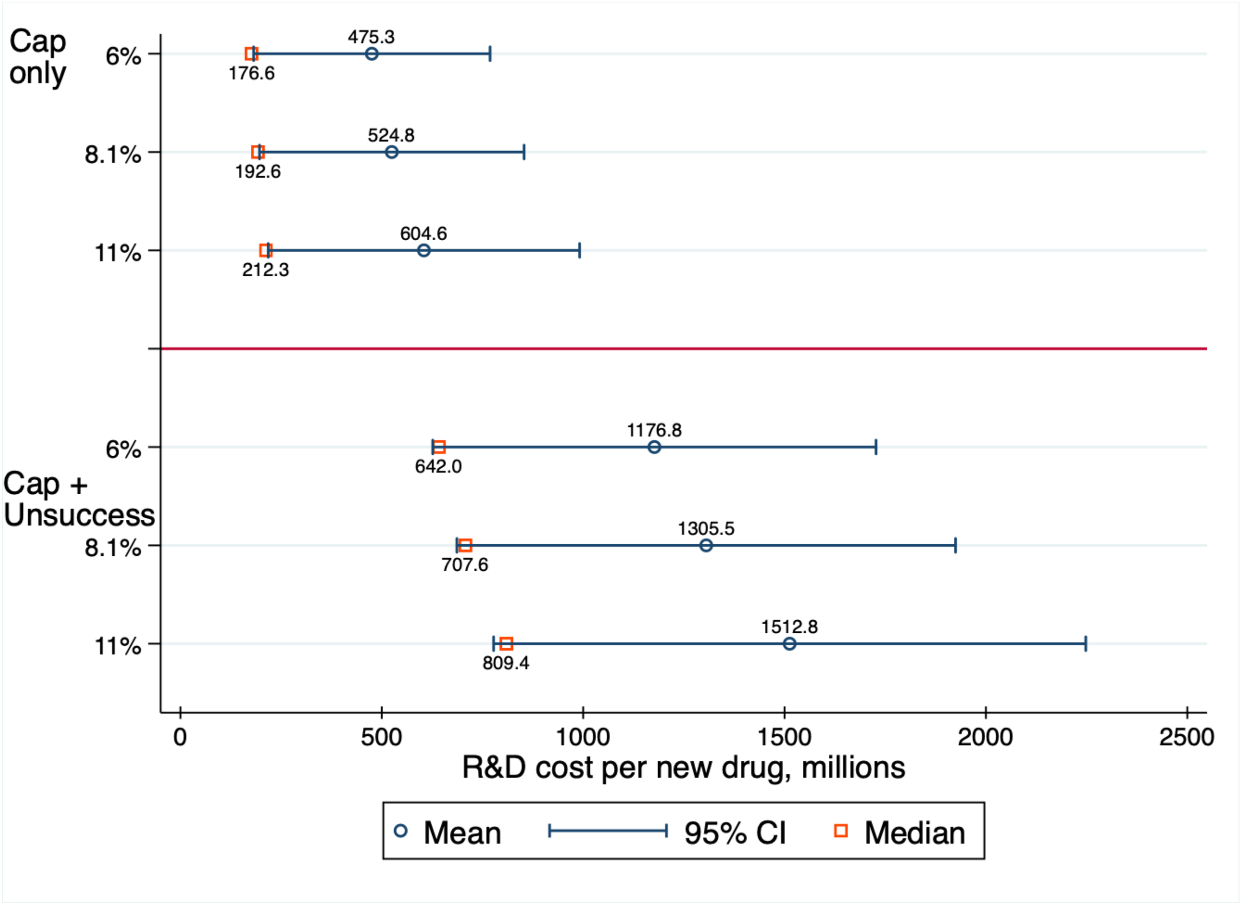

© 2025 Mulcahy A et al. *JAMA Network Open.*

eAppendix 6. Comparison of Trialtrove R&D Activity for 2019 New Drugs to Trials in FDA Review Documents

As a validation check, for 19 of the 38 total 2019 new drugs, we used U.S. Food and Drug Administration (FDA) review and administrative documents accessed via the Drugs@FDA portal to abstract information on the clinical trials used to support initial FDA approval for the cohort of new molecular entities approved in 2019.[7] As part of their review of new drug applications, several types of FDA reviewers (e.g., clinical, pharmacology, statistical) summarize and assess the design and findings from clinical trials described in regulatory filings. The reviews list relevant clinical trials assessed by the FDA and typically describe enrollment and key dates for each trial.

We collected details from FDA reviews including the phase, enrollment, and key dates for each mentioned trial. We calculated patient-months for each trial as the product of enrollment and duration and aggregated patient-months across all trials for a given drug.

We found that clinical trial information is reported in the same level of detail or consistently across applications and individual FDA reviews. More specifically, trial duration was not always reported in the FDA documentation (this was most common for Phase I and II trials). Overall, for each of the 20 drugs compared in this check, Trialtrove had the same number or more clinical trials compared to the clinical trials described in FDA review documents. For a given drug, the surplus Trialtrove trials were for indications other than the initial FDA-approved indication, were co-sponsored with academic collaborators, or had relatively later start dates such that results were likely not available at the time of FDA review (and, later, approval). Because we included all Trialtrove records linked to each drug and sponsor, regardless of indication and

---

[7] FDA. Drugs@FDA: FDA-Approved Drugs. 2023 [cited October 15, 2023; Available from: https://www.accessdata.fda.gov/scripts/cder/daf/index.cfm.

© 2025 Mulcahy A et al. *JAMA Network Open.*

through the FDA approval date, we view our estimate as conservative in the sense that it includes sometimes many trials not considered by the FDA as needed for the basis of an initial approval. While some of the surplus clinical trials included in our estimation later expand the approved indications for a drug, in other cases the later trials serve to support marketing claims, product lifecycle management strategies, and other drug company interests.

© 2025 Mulcahy A et al. *JAMA Network Open.*
