# Supplementary material for: Use of Clinical Trial Characteristics to Estimate Costs of New Drug Development
Source: JAMA Netw Open. 2025 Jan 6;8(1):e2453275. doi: 10.1001/jamanetworkopen.2024.53275 (PMC11704977; doi:10.1001/jamanetworkopen.2024.53275)
Supplement: Supplement 2. — Data Sharing Statement [file jamanetwopen-e2453275-s002.pdf]

Data Sharing Statement

Mulcahy. Use of Clinical Trial Characteristics to Estimate Costs of New Drug Development. *JAMA Netw Open*. Published January 06, 2025. doi:10.1001/jamanetworkopen.2024.53275

Data
Data available: No

Additional Information
Explanation for why data not available: The authors will share (upon request) data abstracted by our study team from publicly available sources (namely, R&D spending information from the U.S. Securities and Exchange Commission). This data is available separately from third-party commercial vendors but at a cost that may be prohibitive to some researchers and projects. The authors will also share (upon request) specific drug-level cost estimates in the Supplement. We cannot share the underlying clinical trial information as we accessed this information under a paid license. The information is available for others to use but at a cost.
